# Health care access from the rural perspective: A narrative review

**DOI:** 10.1111/jrh.70119

**Published:** 2026-02-13

**Authors:** Peter Kaboli, Adam Blaine, Jasmine Mares, John Fortney, Sarah Ono, Amy M. J. O'Shea

**Affiliations:** ^1^ VA Office of Rural Health Veterans Rural Health Resource Center‐Iowa City (VRHRC‐IC), Iowa City Veterans Affairs Health Care System Iowa City Iowa USA; ^2^ Center for Access and Delivery Research and Evaluation (CADRE) Health Systems Research, Iowa City VA Healthcare System Iowa City Iowa USA; ^3^ The Department of Internal Medicine University of Iowa Carver College of Medicine Iowa City Iowa USA; ^4^ Department of Psychiatry and Behavioral Sciences School of Medicine, University of Washington Seattle Washington USA; ^5^ Center of Innovation for Veteran‐Centered and Value‐Driven Care Health Systems Research, VA Puget Sound Health Care System Seattle Washington USA; ^6^ VA Office of Rural Health Veterans Rural Health Resource Center‐Portland (VRHRC‐P) VA Portland Health Care System Portland Oregon USA; ^7^ Center to Improve Veteran Involvement in Care (CIVIC) Health Systems Research, VA Portland Health Care System Portland Oregon USA; ^8^ Department of Psychiatry School of Medicine, Oregon Health & Science University Portland Oregon USA

## Abstract

**Purpose:**

Health care access has been described using several definitions and frameworks, but none are specific to rural populations. We describe ways access to care is measured using the model of access developed by Fortney et al., with a focus on the differential impact on rural populations.

**Methods:**

We describe patient‐centered access metrics in rural and urban populations using the Fortney model's five access dimensions: geographic, temporal, financial, cultural, and virtual. Patient‐level access is put into context of the broader environment in which they live (i.e., their community, health care providers, and health care system).

**Findings:**

Rural populations face similar access challenges as urban residents but are disproportionally impacted by four interrelated challenges: (1) geographic access barriers of distance and available transportation, (2) virtual access care barriers manifested by broadband internet access and digital literacy, (3) health care workforce shortages, and (4) differential impact of social determinants of health. Key facilitators to overcome these include: (1) integrated public transportation services when in‐person care is required; (2) expanded broadband coverage, affordability, and education to ensure access to telemedicine services; and (3) training, incentives, and support for the rural workforce.

**Conclusions:**

Health care systems should incorporate access metrics into routine data collection to drive improvement and ensure equitable access to quality health care. Rural systems of care may be disproportionally impacted by access challenges which require unique approaches for improvement.

## INTRODUCTION

With the implementation of the Affordable Care Act, COVID‐19 pandemic, and technological advances, access to health care has evolved over the past decade. This is especially true in rural settings where 19.3% of Americans reside^1^ and long travel distances significantly influence geographic access to care.^2^, ^3^, ^4^, ^5^, ^6^, ^7^, ^8^, ^9^, ^10^, ^11^ Rural communities were impacted by record closure of hospitals following the end of the CARES Act during the COVID‐19 pandemic.^12^, ^13^ Low to negative operation margins, states’ choice not to not expand Medicaid,^14^ and decreasing Medicare reimbursement have disproportionately affected the ability of rural residents to obtain care.^15^, ^16^ To overcome geographic barriers to care, innovations in virtual medicine have been adopted.^17^, ^18^ Technology has the ability to mitigate geographic access differences due to travel distance, and yet, a lack of broadband infrastructure has impacted rural America diminishing the potential of telemedicine advantages.^19^

Technology alone may not be able to overcome provider staffing shortages in rural communities,^20^ nor the cultural and legislative barriers which make it difficult to attract and retain qualified medical professionals in remote locations.^21^ For the rural aging population who experience increasing health concerns, these factors create a growing challenge to accessing health care,^22^ especially as Medicare projections anticipate increasing spending for the “baby boomer” generation, which is even more concentrated in rural locations.^23^ Thus, rural access metrics must be established to evaluate health care system changes and facilitate future advances in health care access.

Building on previous models,^24^, ^25^, ^26^, ^27^ Fortney et al. constructed a new access framework in 2011, which included five well‐defined dimensions (i.e., geographical, temporal, financial, cultural, virtual).^28^ This framework was appropriately adapted to virtual changes in health care delivery, including asynchronous communication, video telemedicine, and remote monitoring programs and is the framework on which this narrative review is constructed. This model also highlights the critical importance of perceived access in addition to actual access, which was subsequently operationalized and psychometric validated.^29^ For the purposes of this paper, we will focus on actual access.

Within our narrative review, we highlight the unique challenges that must be addressed to improve access for rural populations. Using the Fortney model,^28^ we examine current metrics to measure access and summarize interventions to drive access improvements in rural health care. Although distance to health care facilities will continue to be a primary access barrier, we anticipate innovative solutions to transportation, virtual services, and workforce staffing models can have a positive impact on health care access for rural Americans.

## METHODS

**FIGURE 1:**
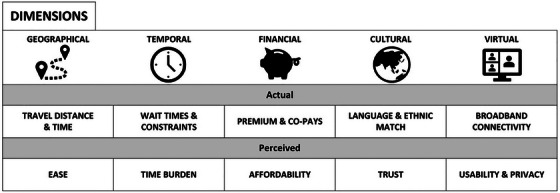
Fortney Access Model. Access represents the potential ease of having virtual or face‐to‐face interactions with a broad array of health care providers, including clinicians, caregivers, peers, and computer applications.

**FIGURE 2:**
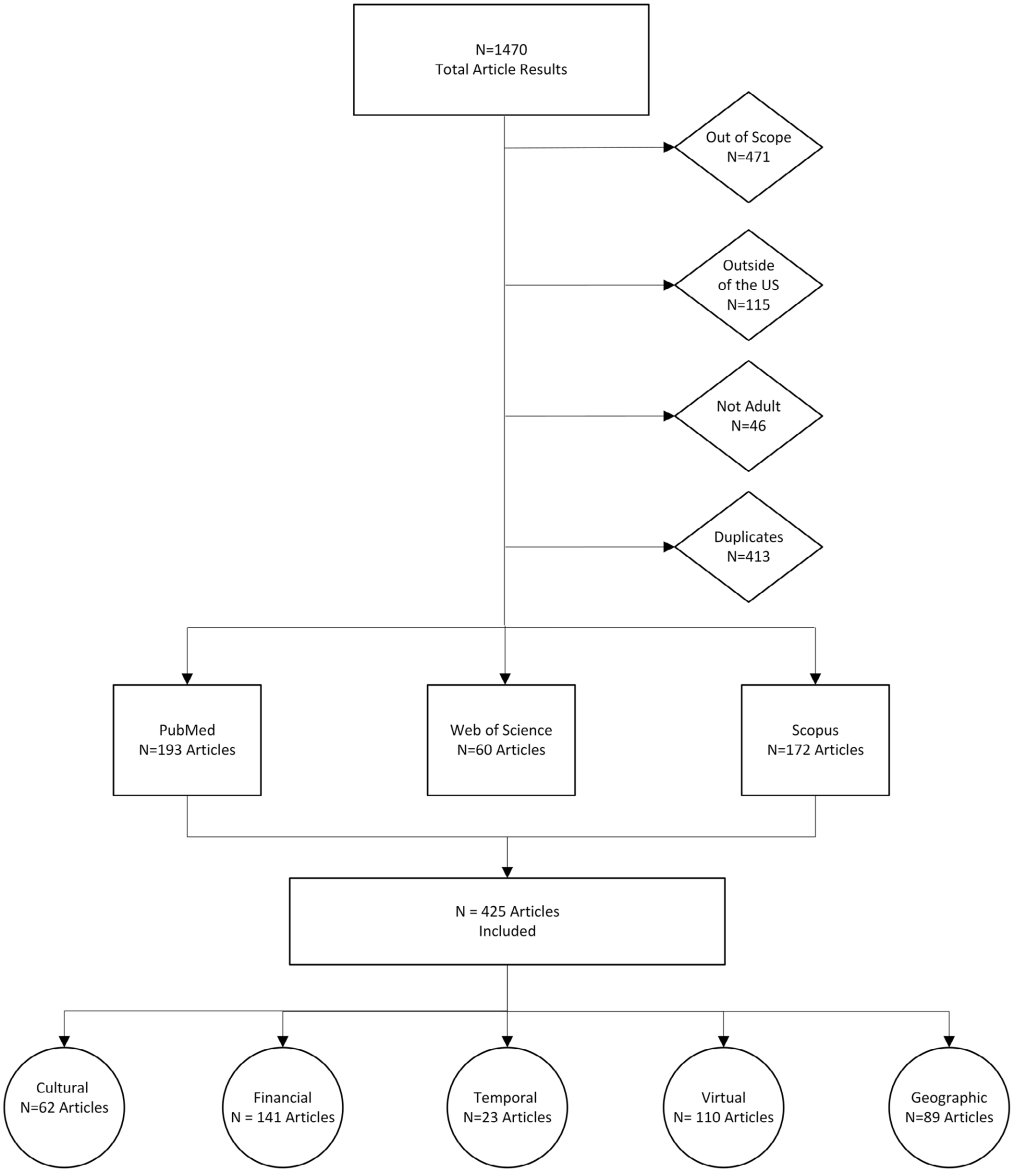
Overview of the literature review process ending in how many publications were found for each dimension of access.

This narrative review involved a comprehensive literature search using the Fortney model and the five dimensions of access with associated metrics to identify relevant literature. Multiple keywords were used for each model dimension (Table 1) and searched in PubMed, SCOPUS, and Web of Science. This targeted search presented publications that were practical to rural populations and related to the Fortney model of access. Literature met the inclusion criteria if it was: (1) published from July 2013 thru July 2023, (2) pertained to or included data about the US population, and (3) was written in the English language. Together this search criteria identified 425 unique journal articles with an updated view of health care access, with specific metrics related to the five Fortney dimensions (i.e., geographic, temporal, financial, cultural, virtual) (Figures 1 and 2).

**TABLE 1:** Search terms used for the Fortney dimensions.

Dimension	Definition	Keywords	Metrics
Geographical	Travel distance/time	distance; time	Travel time and transportation
Temporal	Time to next appointment Reception wait time	time; availability	Time spent waiting in reception, time spent receiving treatment, and time for the next available appointment^42^
Financial	Eligibility Out of pocket costs	costs	Insurance premiums, out‐of‐pocket costs, and cost of digital connectivity^45^
Cultural	Language match, trust	culture; health care;	Language offered, cultural competency, and provider stigma/discrimination
Virtual	Connectivity	telehealth; telemedicine	Includes equipment or access to equipment, patient education, and the availability of broadband internet service.

*Note*: All keyword searches included the terms rural health; access; United States.

## SYNTHESIS OF ACCESS METRICS BY FORTNEY DIMENSIONS

The results of this narrative review, guided by the five Fortney dimensions, identified access metrics in the scientific literature that are directly observable, objectively measurable, reliable, and have good predictive validity with regards to utilization. These metrics can be translated across health care systems and allow for adaptation to accommodate the characteristics of rural residents (Table 2).

**TABLE 2:** Metrics and interventions for rural access dimensions.

Dimension	Metrics	Interventions
Geographical	Travel time Travel distance Public transportation availability Seasonal and weather considerations	Telemedicine Regional public transportation Patient‐centered care Programs to incentivize rural practice
Temporal	Time spent waiting in reception Time spent receiving treatment Third next available appointment^42^ Timely care Same day access	Improved wait time policies Remote triage
Financial	Insurance premiums Out‐of‐pocket costs/co‐pays Cost of digital connectivity^45^	Health navigators Co‐payment programs Alternative health models and payments
Cultural	Language offered Cultural competency Stigma BRFSS NCLASS	Health literacy Programs to confront stigma Interpreter services Women and minoritized group support programs
Virtual	Equipment or access to equipment Patient/caregiver education Availability of broadband internet service.	eHealth literacy Low‐earth orbit satellite internet

Abbreviations: BRFSS, behavioral risk surveillance system; NCLASS, national culturally and linguistically appropriate service standards.

### Geographic

Although rural living has many benefits and rural residents accept and accommodate for geographic isolation, some may not have a choice, the social support, and/or financial means to overcome transportation challenges that geographic barriers pose. Urban populations, for example, generally have a higher density of health care providers, shorter travel times, and more widely available public transportation and ride‐share services.^30^ It is not surprising then that in the literature, geographic access is the most consistently considered distinction between rural and urban populations. Distance and travel time^5^, ^31^, ^32^, ^33^, ^34^ are often assessed, though travel time is preferred due its ability to account for dissimilar terrain (e.g., mountainous) or driving conditions (e.g., traffic congestion). Distance alone, without the inclusion of travel time, can be misleading especially for the most remote locations and dense urban areas. Travel time provides researchers with a simple comprehensive metric that accurately captures the distance‐related challenges faced by rural populations in accessing health care and identifies areas with inadequate geographic access.

Other metrics that are determinants, and therefore correlates, of geographic access include the designation of rural geographic areas or presence of health care providers in a certain area.^31^ Geographic Information Systems (GIS)^30^, ^32^ can be used to link these area‐level metrics (e.g., counties, zip codes) to individuals, health care systems, or communities using latitude and longitude values.^35^ In areas for which current metrics are inadequate to guide decision‐making, spatial modelling methods that better account for mountainous terrain, highly rural areas, or relative socio‐economic scores should be considered. For example, two‐step floating catchment area methods have been applied to measure access to care in Appalachia,^36^ while a modified version was applied to measure health care access in West Virginia.^37^

### Temporal

Temporal access represents time required to receive services and the opportunity cost of that time. Such access is usually described in terms of “wait times,” or the simple calculation from the point when a health care service was requested and when it was met. Wait times have been calculated for specific patient subgroups (e.g., established or new patient), or within different care modalities.^38^, ^39^, ^40^ Importantly, wait times have been found to vary substantially by care category (i.e., primary care, mental health, other specialties) and geographic location.^39^, ^41^ Ultimately, longer wait times can deter patients from seeking care and eventually lead to more complex medical treatment due to delays or more severe symptoms.^42^ For older patients, who disproportionally live in rural areas, wait times can especially be of concern.

### Financial

In the United States, the primary cause of financial barriers is lack of health insurance. This has been improved since the passage of the Affordable Care Act (ACA) in 2010, which expanded insurance benefits to both rural and urban populations. In 2018, roughly 12 million consumers purchased insurance coverage through the ACA Marketplace^43^ adding to the millions of people already on Medicare, Medicaid, and private insurance plans. The number of uninsured Americans has declined by half from 46.5 million in 2010 (15% of the US population) to 25.6 million in 2022 (7.7% of the US population).^44^ Despite expanded financial access, evidence from the federally facilitated and state‐based Marketplaces suggests premiums in rural versus urban counties are higher and have grown faster over time.^43^, ^45^, ^46^, ^47^ With fewer residents in rural areas, insurance companies must charge more to cover costs with fewer participants in the market.

In addition, being “under‐insured” can result in higher copays with devastating out‐of‐pocket costs.^48^ This is especially relevant for rural populations who have fewer options for health plans, lower education levels, and less understanding of insurance markets. Rural populations often struggle when comparing insurance plans and regularly choose plans with higher out‐of‐pocket maximums,^48^ perpetuating medical debt. Lastly, higher travel costs for rural populations further contribute to the expense of attending in‐person appointments.

### Cultural

Rural residents have unique cultural differences and attitudinal barriers that are often overlooked by health care systems developed for the population at large. For example, attitudinal barriers such as stoicism, self‐reliance and feelings of independence among rural residents can impact health care seeking.^49^ Furthermore, attitudinal differences leading to delays in seeking care may lead to feelings of stigmatization when visiting health care providers. This can perpetuate a perception that there is a punitive nature to the visit, whether they had neglected their health or had a lack of health literacy.^50^ This compounds the severity of health conditions, isolating them from their health care provider and further compromising treatment. This social stigma has been measured using Centers for Disease Control (CDC) produced comprehensive community surveys, such as the Behavioral Risk Surveillance System (BRSS).^51^ These comprehensive self‐evaluation surveys evaluate disparities in health, access to care, and health related behaviors.^51^ These random phone interview surveys offer a tool for rural residents to communicate health‐related concerns and determine barriers to health care. In addition, the National Culturally and Linguistically Appropriate Service Standards (NCLASS), previously backed by the US Department of Health and Human Services, is a set of 14 steps intended to advance health equity, improve quality, and eliminate health care disparities, with the potential to help evaluate cultural components of health care access.^51^ These thoughtfully designed surveys could be integrated into a scoring system to evaluate the cultural aspect of rural access. However, the authors acknowledge cultural access is the hardest dimension to measure and may be better suited to metrics of *perceived* access.

### Virtual

Accessibility from a virtual standpoint considers the remote connectivity to providers, whether in real time or asynchronously, across virtual communication platforms. During the COVID‐19 pandemic while in‐person visits were restricted, the virtual dimension of health care rapidly expanded, broadening the opportunity for rural access. Among rural populations, those familiar with the internet and virtual technology (e.g. computers, tablets, smartphones) were quick to transition to the virtual environment, while older patients and people with poor connectivity and poor digital literacy struggled to adapt.^52^ Though virtual health services have the potential to decrease costs and expand primary care and specialty services to rural locations, patient buy‐in and associated broadband internet and computer costs remains an obstacle contributing to the “digital divide.”^53^

Telephone surveys have been used to measure elements of the virtual dimension. These surveys offer the ability to assess patient access to virtual technology, internet access, history of using the internet for communication, and telehealth availability with their health care provider.^52^ However, like the cultural dimension that is dependent on how questions are asked and how respondents reply, telephone surveys may reflect *perceived* rather than actual access. Additionally, research has found broadband internet access is a significant barrier for rural, underserved populations.^19^, ^54^, ^55^ Without the availability of reliable and sufficiently fast internet to support use of video (a social determinant of virtual access), rural patients cannot take full advantage of the benefits of telemedicine. For example, rural veterans experienced lower increases in the use of video visits during the COVID‐19 pandemic than urban veterans, likely due to lower availability of broadband at home.^56^ To measure broadband availability, patient‐level latitude and longitude address can be compared to census block‐based broadband availability using FCC Fixed Broadband data.^19^ However, this metric reflects aspects of the community rather than the patient who may not be able to afford broadband and also excludes satellite internet. Other metrics of determinants of actual access could include whether a health care provider in the patient's network could provide care by interactive video, or proportion of eligible providers willing to do so.

## INTERVENTIONS TO OVERCOME ACCESS BARRIERS

The difference between access barriers and attitudinal barriers, such as stoicism and self‐reliance, are that access barriers are potentially modifiable by the health care system and/or the payer.^29^ Therefore, health care systems and payers should be held accountable to gaps in access and identify strategies to overcome those gaps. Using the above‐mentioned metrics, we propose interventions to improve rural access and provide more geographically equitable care. These interventions, discussed below and in Table 2, should target determinants of access at a variety of levels, including the patient, provider, health system, payer, and/or the community.

### Interventions to overcome geographic access barriers

1. Telemedicine is a great equalizer for overcoming distance, with two important caveats. First, users (e.g., patient, caregiver, providers, health care system) need sufficient broadband access, proper equipment, and the knowledge, skills, and desire to use telemedicine (see ehealth literacy). Second, billing, reimbursement, and licensing policies must be friendly to its usage and consistent across states and insurance programs.^57^ Despite these challenges, since the COVID‐19 pandemic, telemedicine has been widely adopted^58^, ^59^ with both patients and providers expressing satisfaction.^60^, ^61^ Importantly health care systems should carefully consider strategic and appropriate telemedicine utilization. For example, the equivalence of mental health care delivered by telemedicine compared to face‐to‐face has been well established across diagnoses and psychiatric disciplines and is thus a highly viable care modality in this setting.^62^ As health care systems incorporate telemedicine, they should consider current and future infrastructure needs, the scalability to other settings, and the balance between startup costs and return on investment.^63^ Notably, there is some evidence that telemedicine can reduce costs, especially as the distance between patient and provider increases.^64^, ^65^
2. Regional public transportation is necessary for those without means of transportation (e.g., personal vehicle). Unfortunately, rural transportation networks are limited by lack of public funding and/or inadequate demand to make them financially feasible, and yet their impact can be profound especially for older rural residents who are no longer able to drive themselves. The Veterans Health Administration (VHA) has partially overcome these transportation barriers through the Veterans Transportation Service (VTS) and Veteran Service Organization van services (e.g., American Legion, Disabled American Veterans). To replicate this success, peer‐to‐peer networks could facilitate ride sharing, coordinate volunteer drivers and build social cohesion especially if paired with community‐based organizations. Alternatively, health care systems could consider starting their own affordable transportation services. Transportation could also be combined with telemedicine to decrease drive distance and minimize costs.
3. Patient‐centered care focuses on good communication between patients and the health care system. It is critical when geographic access barriers exist. Although it may be routine to schedule in‐person appointments to review diagnostic testing or facilitate prescription refills, follow‐up by telephone may achieve similar results.^66^ Likewise, the use of electronic‐consults from primary care to specialists may answer the necessary clinical question or facilitate additional work‐up closer to the patient's home, avoiding unnecessary travel (e.g., pre‐operative evaluations, sub‐specialty care).^67^ When in‐person visits are necessary, schedulers could use “visit chaining” to minimize the need to make multiple trips to the clinic.^68^
4. Programs to incentivize rural practice can improve access to clinical services (e.g., primary care, mental health) and reduce wait times. Incentive programs, including loan repayment, educational opportunities, and recruitment or retainment bonuses should be offered to providers, nurses, and other health care workers dependent on local needs. Further to increase the health care workforce in rural settings, schools and training programs must promote careers in demand in rural areas (e.g., physicians, nurse practitioners, physician assistants, nurses, nursing assistants)^69^ and expose students to providing care in rural locations. Medical schools have successfully incorporated rural rotations into their training programs, implemented focused rural admissions processes, and financially supported students who choose rural medicine.^70^, ^71^ Similar to investments made by the VHA, other health care systems should consider funding rural professional development opportunities, implementing communities of practice, and providing interprofessional training to address concerns of professional isolation and scopes of practice.^72^, ^73^, ^74^, ^75^, ^76^

### Interventions to overcome temporal access barriers

1. Improved wait time policies require collecting wait time data and implementing programs to reduce wait times to an agreed upon industry‐wide standard. No such standard currently exists and may not be easily defined across states, health care systems, or specialties given disparate local geographies, patient mix, and provider availability. For example, within Medicaid, there is wide variability in how states define time or distance access standards, and in how such standards are monitored.^77^ Regardless of the standard applied, multiple interventions can be implemented to reduce wait times, including open‐access scheduling, a primary care gatekeeper model with a cap on provider panel size, and performance monitors to ensure clinics are meeting targeted wait times.^78^
2. Remote triage, usually by telephone, has been used successfully to ensure an appropriate “wait time” to meet the clinical and personal needs of the patient.^79^ Staffed by nurses and often augmented by licensed independent practitioners, these call centers incorporate decision making software with clinical expertise to determine the acuity (e.g., emergent, urgent, routine) and the most appropriate location of care (e.g., emergency department, urgent care, clinical appointment, or telemedicine follow‐up) to meet the patient's need.^80^, ^81^ Remote triage not only helps people receive the right care at the right time, it also allows for the expansion of health care services to rural populations, ensures appropriate resource use, and can reduce downstream health care needs.^80^ A remote telephone triage study of more than 3000 patients resulted in an urgent care length of stay decrease from 2 to 2.5 h before implementation to 35–40 min post‐implementation by consulting with patients at home and directing them to the most appropriate level of care.^82^

### Interventions to overcome financial access barriers

1. Health navigators have been employed successfully by the ACA to facilitate insurance enrollment ^83^ and within oncology programs.^84^ These services can be provided remotely, thus requiring less local infrastructure in rural communities. However, navigators local to the community can better understand local care environments.^85^, ^86^ For example, Community Health Representatives assist with transportation, prescription delivery, and other health‐related services among American Indian residents of the Southwestern United States,^87^ while Veteran Service Organizations, State, and County Veterans Service Officers play a key role in the VHA enrollment process, especially in rural areas. The challenge of incorporating health navigators, particularly in rural areas, is staffing costs. One cost‐effective solution is to integrate education, networking, and advocacy training specific to patient needs within their clinical setting for existing nurses or support staff which would be promoted by the clinic directly to the patient. Such training would require buy‐in from clinic leaders but may be justified by offsets in reduced care fragmentation and improved physician workload.
2. Copayments, a fixed amount of money paid by the patient for health care services, are a common practice. Though this makes the costs of care predictable, these costs can add up for those on limited income or with complex medical conditions requiring frequent visits. In some instances, funding for community health centers via the Patient Protection and Affordable Care Act or grant programs to fund copayment‐free care could increase access.^88^, ^89^, ^90^ Additionally, insurance companies and public policy makers should consider the impact of removing copayments for high‐value care that decreases subsequent costs (e.g., cancer screenings).^91^
3. Many beneficiaries of social insurance programs, including Medicare and Medicaid, live in rural or medically underserved areas with notable shortages of primary care and other providers. Alternative health models and partnerships offer a possible way to address barriers to using existing insurance coverage (e.g., Medicare, Medicaid) by forming innovative partnerships across health care facilities. For example, the mega community health center (CHC) model incorporates neighborhood outreach clinics, a hub CHC, and a teaching hospital to facility specialty care needs.^92^ Federally qualified health centers (FQHC), which serve a high proportion of dual‐eligible patients, could be strategically expanded in high need, rural areas.^93^ Payment reforms that improve insurance coverage levels may also improve the financial viability of rural hospitals,^14^, ^94^ though this would require political motivation to facilitate. Other models could expand the use of midwives, nurse practitioners, or other community health workers in coordination with physicians as warranted or collaboratively sharing physicians within a medical community.^95^

### Interventions to overcome cultural access barriers

1. Health literacy challenges are common in health care, but interventions to address it are limited. Routine health literacy assessments are a way to measure a patient's literacy,^96^ but these assessments add additional burden to health care staff and are not unanimously deemed necessary. Instead, education materials should be written from the patient‐perspective, culturally appropriate, and standardized not to exceed a sixth‐grade reading level to ensure patient relevance and understanding.^97^, ^98^, ^99^ Pamphlets, fliers, or online educational materials complemented by one‐on‐one or group education opportunities, as well as community outreach, have also been endorsed, but how these strategies would be funded remains problematic.^86^, ^100^
2. Stigma, defined as negative perceptions and behaviors for a particular circumstance, quality, or person, can manifest from the public, providers, or self resulting in cultural barriers affecting health care use whether it is enacted (i.e., discrimination) or perceived during care.^101^, ^102^ This enacted or perceived stigmatization can be associated with specific health care conditions (e.g., HIV, hepatitis C, obesity, substance abuse, mental illness), rural residence, or other socioeconomic factors. While health care systems can do little to directly address self‐stigma and public stigma, strategies to reduce provider stigma typically include education, partnership building with patients (i.e., “working alliance”), collaboration with community organizations, and cultural humility.^86^, ^103^, ^104^
3. Interpreter services improve communication when a patient and provider do not share a common language. Lack of interpreters or use of informal or untrained interpreters (e.g., family members) can impact patient safety and satisfaction,^105^ as miscommunication can be life threatening^106^ and frustrating,^107^ especially with growing cultural and linguistic diversities. Linguistic services, virtual or in‐person, can be used to effectively overcome language barriers, though interpretation quality, especially of medical or genetic terms or by speakers from different countries or regions.^108^ Insufficient funding or interagency competition for interpretive resources, especially in underserved or rural areas, can lead to a lack of interpretive services.^109^
4. Women and historically minoritized groups in rural communities can experience health care barriers due to prejudice or discrimination.^110^ Importantly, research has shown women often feel disregarded or diminished by specialty care providers and report that their pain is dismissed, suggesting possible unintended or unconscious gender biases.^111^ These barriers can vary between individuals and change over the life span.^112^ Rural health care services should allow patients to choose gender concordant providers when feasible,^100^ incorporate population relevant traditional healing,^113^ and foster strong relationships with local community organizations to build trust and culturally appropriate care.^90^, ^113^ Cultural competency training may also help medical professionals respectfully interact with others from diverse backgrounds.

### Interventions to overcome virtual access barriers

1. eHealth literacy is an important skillset to engage with secure messaging, interactive video, online patient portals, and health‐related applications.^114^ Health care systems may consider implementing educational interventions, similar to the VHA digital divide consult^19^ or virtual health resource centers (i.e., a walk‐in, in‐person clinic offering support for virtual care tools),^115^ that could guide patients to resources available in their community through public libraries, senior centers, non‐profits, federal and state government, or other organizations. Regardless of the source, training interventions should be tailored to specific populations (e.g., elderly, specific diagnosis groups),^116^ offer multiple learning modalities (e.g., visual, audio, written, or a combination), and meet patients where they are.^117^, ^118^
2. Low‐earth orbit satellite internet is now widely available and unlike terrestrial wireless internet is currently more accessible in rural or geographically remote locations. Though this technology offers a promising alternative to address disparities in terrestrial broadband access, it remains susceptible to high start‐up costs, weather sensitivity, reliability issues, and lack of access to local technicians for installation and ongoing support.^119^ The impact of these barriers may decrease as competition from multiple LEO providers and expansion among end users increases.

## DISCUSSION

Theories of access can be used to understand unique barriers to care faced by rural populations. These communities grapple with significant challenges, particularly geographical and virtual access which are exemplified by long travel distances and limited technological infrastructure. To understand and monitor this, standardized measures tailored to evaluating rural access are imperative. By doing so, we can enhance rural health outcomes while improving geographically equitable care delivery. This systematic review process not only identified metrics and interventions in rural health care access but highlighted four inter‐related health care challenge categories: geography, workforce, the digital divide, and social determinants of health and access.

How we measure access is important. For example, during our search process it was clear the geographic dimension had been most widely used metric of actual access in the rural setting with standardized measures that are accurate and reliable. However, many actual access measures remain understudied (e.g., cultural) or are evolving (e.g., virtual). How actual access is measured has practical implications for how health care systems, payers, and legislators implement initiatives aimed at improving access. Similarly, the authors advocate for a patient‐centric consideration of all access metrics. Patients likely weigh each dimension of access based on their priorities and preference when making decisions about seeking treatment. For example, if two health care systems each have long wait times, patients may balance the need for a timely visit against the added burden of distance or the disparate costs for going “out of network.”

Geographically remote and isolated locations continue to suffer from an inability to fully join the virtual age. Low‐earth orbit satellites are a key alternative to costly and time‐intensive infrastructure projects to provide terrestrial broadband nationwide and offer a long‐term solution to areas with low subscription rates. As this barrier is overcome, downstream effects on access measured within the temporal and financial domains can also be improved via telehealth visits, electronic consults, improved provider‐patient asynchronous communication, and remote triage of care. Cultural access barriers might be the most difficult dimension of actual access to measure and address. However, telehealth can overcome some health literacy barriers, improve social support and education to decrease provider stigma, and facilitate access to interpreter services.

This review has several limitations. First, measuring actual cultural barriers remains elusive, necessitating further exploration and greater reliance on perceived measures. Current techniques are subjective and inconsistent across multiple health care settings, especially with regards to primary data collected via survey. Second, data from rural populations is often scarce due to poor health system inter‐connectivity when compared to large‐scale urban hospital systems. While the VHA system boasts a robust network of hospitals, smaller rural health care systems (e.g., FQHCs, Rural Health Clinics, Critical Access Hospitals, Rural Emergency Hospitals) serving rural communities typically do not have strong data collection while also facing implementation and funding challenges.

In conclusion, improving access for rural populations holds immense potential and addressing geographical and virtual barriers to health care may have the greatest short‐term potential for closing the urban–rural inequities in access. Overall using an access framework of dimensional metrics and mapping them on to interventions, future studies and innovations can target goals based on characteristics of quality, outcomes, utilization, and satisfaction to improve barriers to access in rural areas. After all, we cannot improve what we are unable to measure. Future work must use consistent, patient‐centered measures so interventions can be appropriately targeted.

## CONFLICT OF INTEREST STATEMENT

The authors declare no conflicts of interest.

## DISCLOSURES

The views expressed in this article are those of the authors and do not necessarily represent the views of the Department of Veterans Affairs or the United States Government. The authors report no conflict of interest regarding this study.
